# Investigation into the association of *FNDC1* and *ADAMTS12* gene expression with plumage coloration in Muscovy ducks

**DOI:** 10.1515/biol-2022-0877

**Published:** 2024-06-11

**Authors:** Guo-Bo Sun, Yan-Feng Lu, Xiu-Jun Duan

**Affiliations:** Animal Science and Technology College, Jiangsu Agri-animal Husbandry Vocational College, Taizhou, 225300, Jiangsu, China; Animal Science and Technology College, Jiangsu Agri-animal Husbandry Vocational College, No. 8 of Fenghuang East Road, Hailing District, Taizhou, 225300, Jiangsu, China

**Keywords:** *ADAMTS12*, feather color, *FNDC1*, Muscovy duck, protein expression, RT-PCR

## Abstract

To elucidate the molecular genetic mechanisms underpinning feather color in Muscovy ducks. A cohort of 100 Muscovy ducks was meticulously selected for this research. Follicular tissues from ducks exhibiting black and white plumage served as the experimental samples. From these tissues, RNA and proteins were extracted for further analysis. The RNA underwent reverse transcription polymerase chain reaction amplification, followed by validation through western blot assays. The data revealed a significant upregulation in the expression of FN domain-containing protein 1 (*FNDC1*) and *ADAMTS12* genes in Muscovy ducks with white plumage traits as opposed to those with black plumage traits. Specifically, individuals with pure white plumage demonstrated a markedly elevated expression of the *FNDC1* gene in comparison to their pure black counterparts. Conversely, expression levels of the *ADAMTS12* gene were found to be reduced in ducks with pure black plumage relative to those with pure white plumage. Notably, the expression patterns of *FNDC1* and *ADAMTS12* genes exhibited inconsistencies between mRNA and protein levels. This study offers significant insights into the molecular genetic mechanisms underlying feather color variation in Muscovy ducks. *FNDC1* and *ADAMTS12* could be considered potential targets for genetic manipulation or selective breeding strategies aimed at achieving specific feather color phenotypes in Muscovy ducks.

## Graphical abstract

**Figure:**
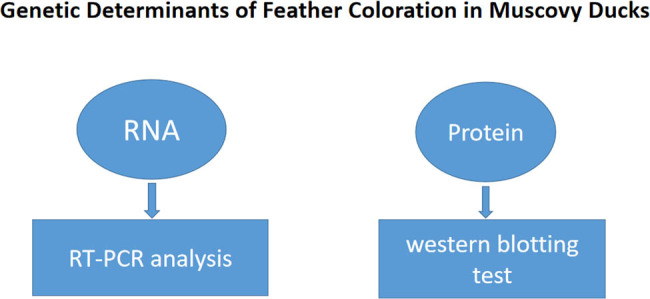

## 1 Introduction

The Muscovy duck (*Cairina moschata*), native to South and Central America, has been introduced to numerous countries worldwide, including China, over the past two centuries [1]. Distinguished from other duck varieties by its unique attributes, the Muscovy duck is characterized by low body fat, a high proportion of lean meat, robust resilience to nutritional challenges, formidable disease resistance, and superior liver quality in comparison to other poultry breeds [2]. The notable characteristics of the Muscovy duck underscore its significant potential for future development within avian agriculture.

Feather color, serving as a critical genetic marker in ducks, constitutes a readily apparent epigenetic trait [3]. Studies in this field have demonstrated that the genesis of duck feather coloration is principally governed by melanin production, which is subject to the influence of several genes [3,4]. Classical genetic models on duck feather color suggest that nine loci are instrumental in this process, interacting synergistically to manifest a spectrum of feather colors [4,5]. Historically, research efforts have primarily concentrated on the genetic inheritance mechanisms of white feather traits [5]. However, the breeding and selection processes for Muscovy ducks are intricate and the existing methodologies are not without flaws. Therefore, it is essential to undertake additional research focused on the genetic mechanisms regulating the black feather gene in Muscovy ducks, to enhance breeding strategies and selection criteria.

In this study, Muscovy ducks exhibiting three unique feather colors – black, white, and floral – were selected. Sample collection occurred at two distinct developmental stages: 120 and 300 days. Following established protocols, tissue RNA and proteins were extracted. The mRNA was then converted into complementary DNA (cDNA), serving as a basis for designing primers for reverse transcription quantitative polymerase chain reaction (qRT-PCR). This technique was employed not only to validate sequencing outcomes but also to evaluate RNA purity and to facilitate the development of antibodies for subsequent western blot verification experiments.

The FN domain-containing protein 1 (*FNDC1*) and *ADAMTS12* genes have garnered significant interest within the realm of biological research. The protein product of the *FNDC1* gene is a transmembrane protein, which exhibits widespread expression across diverse cell types. Notably, *FNDC1* has been associated with the pigmentation of skin and feathers in animals, particularly in avian species. The expression level of the FNDC1 gene has been found to have a direct correlation with the coloration and patterning of feathers in birds.

Likewise, the protein produced by the *ADAMTS12* gene is implicated in the color traits of animals. As a metalloproteinase, *ADAMTS12* is involved in the metabolism and regulation of various extracellular matrices. Its critical function in modulating the synthesis and degradation of collagen has a significant impact on the color and texture of animal skin and feathers. A thorough investigation into these two genes can aid in uncovering the molecular mechanisms behind pigment deposition and coloration in animals, while also offering insights into their significance in evolutionary processes and natural selection.

Our research centered on the *FNDC1* and *ADAMTS12* genes, exploring their association with the feather color phenotype in Muscovy ducks. The objective was to decipher the genetic determinants influencing feather coloration in this species, thereby laying the groundwork for precision breeding strategies aimed at cultivating Muscovy ducks with a diverse palette of feather colors. The insights garnered from this study are intended to facilitate the identification of specific loci or genes that regulate feather color in Muscovy ducks, enhancing the comprehension of the genetic architecture underlying feather color variations in these birds.

## 2 Research material and method

### 2.1 Material

#### 2.1.1 Formation of experimental groups

One hundred Muscovy ducks, displaying an array of feather colors – specifically white and black – were meticulously selected to constitute the experimental group. These ducks were reared from the brooding stage up to the initial egg-laying phase, concluding at 350 days of age. Initially, they were kept on the ground during the brooding period, subsequently transitioning to cage rearing during the breeding phase.


**Ethical approval**: The research related to animal use has been complied with all the relevant national regulations and institutional policies for the care and use of animals.

#### 2.1.2 Experimental instruments and reagents

The experimental setup encompassed a comprehensive suite of instruments and reagents, including: Trizol reagent, chloroform, isopropyl alcohol, 75% ethanol, diethyl pyrocarbonate (DEPC)-treated water, 0.5× tris-borate-EDTA (TBE) buffer, agarose gel, lysate, Oligo dT(18), 40 u/μL RNase inhibitor, 5× reaction buffer, 10 mM deoxy-ribonucleoside triphosphates (dNTPs) mix, M-MuLV reverse transcriptase, 2X real-time PCR master mix, protein powder, and polypropylene gel. The array of equipment used in the study comprised a centrifuge, nano-spectrophotometer, PCR amplification system, electrophoresis apparatus, and PVDF membrane.

### 2.2 Hair follicle sampling of Muscovy duck tissue

For the collection of hair follicle samples, two principal methodologies were utilized: destructive sampling and non-destructive sampling. The non-destructive approach is generally favored due to its rapid and uncomplicated procedure, minimal harm to the animals, and a lower risk of inducing stress in the subjects.

#### 2.2.1 Destructive sampling

To collect skin hair follicle samples from Muscovy ducks, the following procedure was implemented.

First, an area approximately 4 cm^2^ on the side of the duck’s neck was cleaned with a cotton ball soaked in alcohol, ensuring the removal of any superficial feathers. When fuzziness became evident around 10 days later, surgical scissors were carefully used to trim it, followed by another cleansing with an alcohol-soaked cotton ball. Using forceps to lift the skin, a 1 cm^2^ piece of skin was excised from the cleaned area. This skin sample was then placed into a 1.5 mL centrifuge tube filled with PBS and DEPC-treated water, and subsequently stored in liquid nitrogen for later analysis. This process also involved measures to reduce inflammation and promote hemostasis at the site of the sample collection.

#### 2.2.2 Non-destructive sampling

The procedure for collecting feather samples from the left wing of the Muscovy duck involved the following steps.

Initially, feathers that had grown to approximately 5 cm in length and displayed visible content at the root were selected for plucking. This process of plucking and collecting samples was carried out over a period of approximately 20 days. During each session of sample collection, the surface dirt on the feathers was removed using a cotton ball soaked in alcohol. Subsequently, forceps were employed to securely grasp the base of each feather, facilitating the swift extraction of the hair follicles.

For sample preparation prior to analysis, 0.5 cm segments of the hair follicles were carefully trimmed and immersed in PBS for thorough cleaning. Subsequently, these cleaned samples were promptly placed in liquid nitrogen for preservation through freezing. The procurement of hair follicle tissue samples was systematically executed across the three distinct feather color categories of Muscovy ducks at two developmental milestones: 120 and 300 days. Notably, the samples were derived from specific areas on each duck that exhibited significant color transitions in their plumage, particularly from black to white, to closely study the genetic underpinnings of feather color variation.

### 2.3 Sample RNA extraction

The RNA extraction from hair follicle tissues was conducted using the Trizol method, a technique known for preserving RNA integrity. Initially, samples were pulverized in liquid nitrogen and homogenized in 1 mL of Trizol reagent, followed by a 5 min incubation at room temperature. The addition of chloroform preceded centrifugation, after which the supernatant was mixed with isopropyl alcohol. A subsequent centrifugation step allowed for the RNA precipitate to be collected, which was then washed with 75% ethanol and left to air-dry. Finally, DEPC-treated water was used to dissolve the RNA, which was thereafter stored at −80°C for preservation. It is important to note that while Trizol, a phenol-based reagent, is effective in ensuring the integrity of RNA during the processing, it requires careful handling due to its potential health hazards.

### 2.4 Agarose gel electrophoresis

To conduct the electrophoresis, the tank and gel plate were first set up. Then, a 6 μL aliquot of the previously described RNA sample was thoroughly mixed with 0.5× TBE buffer. This mixture was subsequently loaded into the gel specimen port. Upon turning on the power switch, the voltage was adjusted to 6 V/cm. The electrophoresis was performed, allowing the RNA to migrate from the negative electrode toward the positive electrode over a span of approximately 25 min. Finally, the results were examined using a UV transmission detector.

### 2.5 Determination of RNA concentration and purity

To evaluate RNA purity and integrity, the NanoPhotometer P 360 (Implen, Germany) was utilized. Initially, the spectrophotometer’s detection base was calibrated through cleansing with DEPC-treated water. For zero calibration, a 1 μL droplet of DEPC-treated water was applied, followed by drying the surface using mirror wiping paper. Afterward, a 1 μL aliquot of the RNA sample was deposited on the sample base, and the sample arm was lowered to initiate analysis. Post-analysis, the arm was meticulously cleaned with dust-free paper to eliminate any residual sample, maintaining the instrument’s precision for subsequent tests. The RNA concentration was determined, and sample purity was assessed based on the A260/A280 absorbance ratio. Given that proteins absorb at 280 nm, a pure RNA sample is expected to exhibit a ratio greater than 2.0. Ratios falling below 2.0 suggest the presence of impurities, indicating compromised RNA purity.

### 2.6 RT-PCR detection and RNA reverse transcription

The initial specimen for the RT-PCR analysis comprised RNA extracted from the hair follicles of Muscovy ducks, with β-actin serving as the internal control. To construct a cDNA library, the Illumina mRNA-Seq 8 sample kit was utilized. Based on the cDNA and protein data, specific RT-q-PCR primers were designed (as listed in Table 1). For the reverse transcription process, 4 μL of total RNA and 2 μL of Oligo dT (18) were combined in a microcentrifuge tube. This mixture was then incubated at 65°C for 5 min, followed by a 5 min cooling period on ice. Subsequent additions included RNase inhibitor, reaction buffer, dNTPs, and M-MuLV reverse transcriptase. The synthesized cDNA was preserved at −20°C. The final reaction mixture totaled 20 μL, comprising 10 μL of 2X real-time PCR master mix, 1 μL of the cDNA template (diluted tenfold), 2 μL of primer MIX (with forward and reverse primers at 10 μM each), and 7 μL of 0.1% DEPC-treated water. The PCR protocol included an initial denaturation step at 95°C for 5 min, annealing at 60°C for 20 s, and extension at 72°C for 40 s, across 40 cycles.

**Table 1:** Primer sequences associated with feather color of black Muscovy duck

Gene name	Primer
β-actin	F:CTCTGACTGACCGCGTTACT
	R:TACCAACCATCACACCCTGAT
FNDC1	F:GGAAGGGGTGAAGACCACTG
	R:GGCGGTAATACCCTCGGATG
ADAMTS12	F:AAAAGCCGGATCATGCCAGA
	R:CAGTGCACTCATTCCAAGGTTC

### 2.7 Protein extraction and western blotting verification test

Fresh muscle tissue from a Muscovy duck was minced and placed into a centrifuge tube, which was then completely filled with protein grinding powder and lysate. Using a plastic pestle, the tissue was pulverized for approximately 2–3 min. More lysate was added, and the grinding process was extended for an additional minute to ensure thorough homogenization. Following this, the tube was centrifuged at maximum speed for 1 min to separate the supernatant, which contained the extracted proteins, from the tissue debris. This supernatant was then carefully decanted into a new tube for subsequent analysis.

Protein analysis was conducted using western blotting. Post-separation, the proteins were transferred onto a PVDF membrane to facilitate detection. This detection involved the application of specific primary antibodies that bind to the target protein, followed by secondary antibodies that bind to the primary antibodies. The proteins were then evaluated based on their resultant coloration, which indicates the presence and abundance of the target protein within the sample.

### 2.8 Statistical analysis

Statistical analysis was performed using GraphPad Prism software. Data are presented as mean ± standard deviation. One-way analysis of variance followed by Tukey’s *post hoc* test were employed to determine significant differences among multiple groups. *p* < 0.05 was considered statistically significant. All experiments were conducted in triplicate, ensuring reliable and reproducible results.

## 3 Results

### 3.1 Sample QC data (agarose gel electrophoresis)

**Figure 1:**
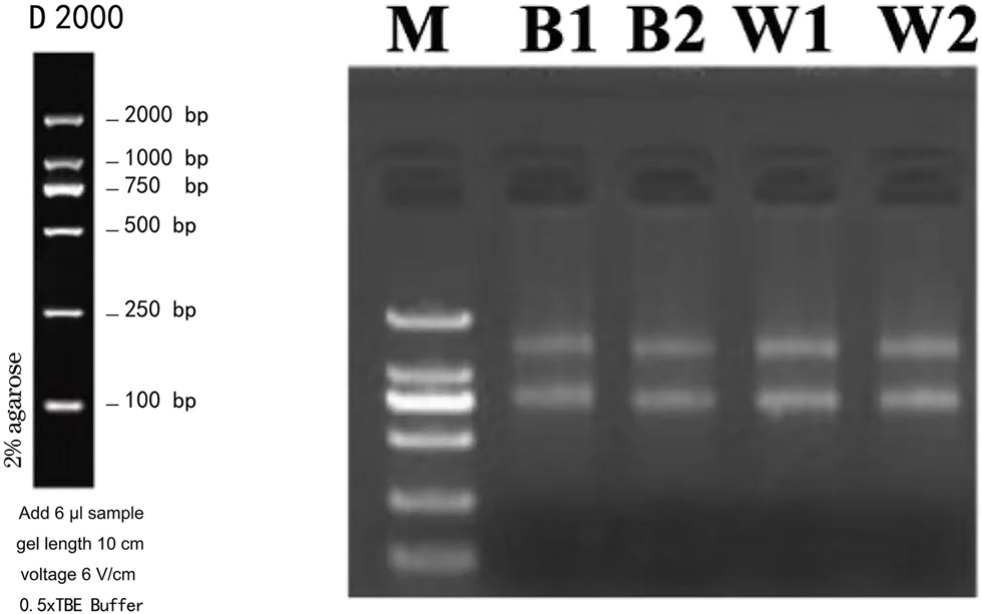
Results of agarose gel electrophoresis. Note: gel plots: Maker: 3 μL, sample: 150 ng; M: Maker; B: black; W: White.

In this article, we detail the evaluation of RNA samples utilizing PCR and agarose gel electrophoresis, methods that successfully met the established testing criteria. The comprehensive results of this assessment are depicted in Figure 1.

### 3.2 Expression results of the *FNDC1* and *ADAMTS12* genes

#### 3.2.1 mRNA expression results

**Figure 2:**
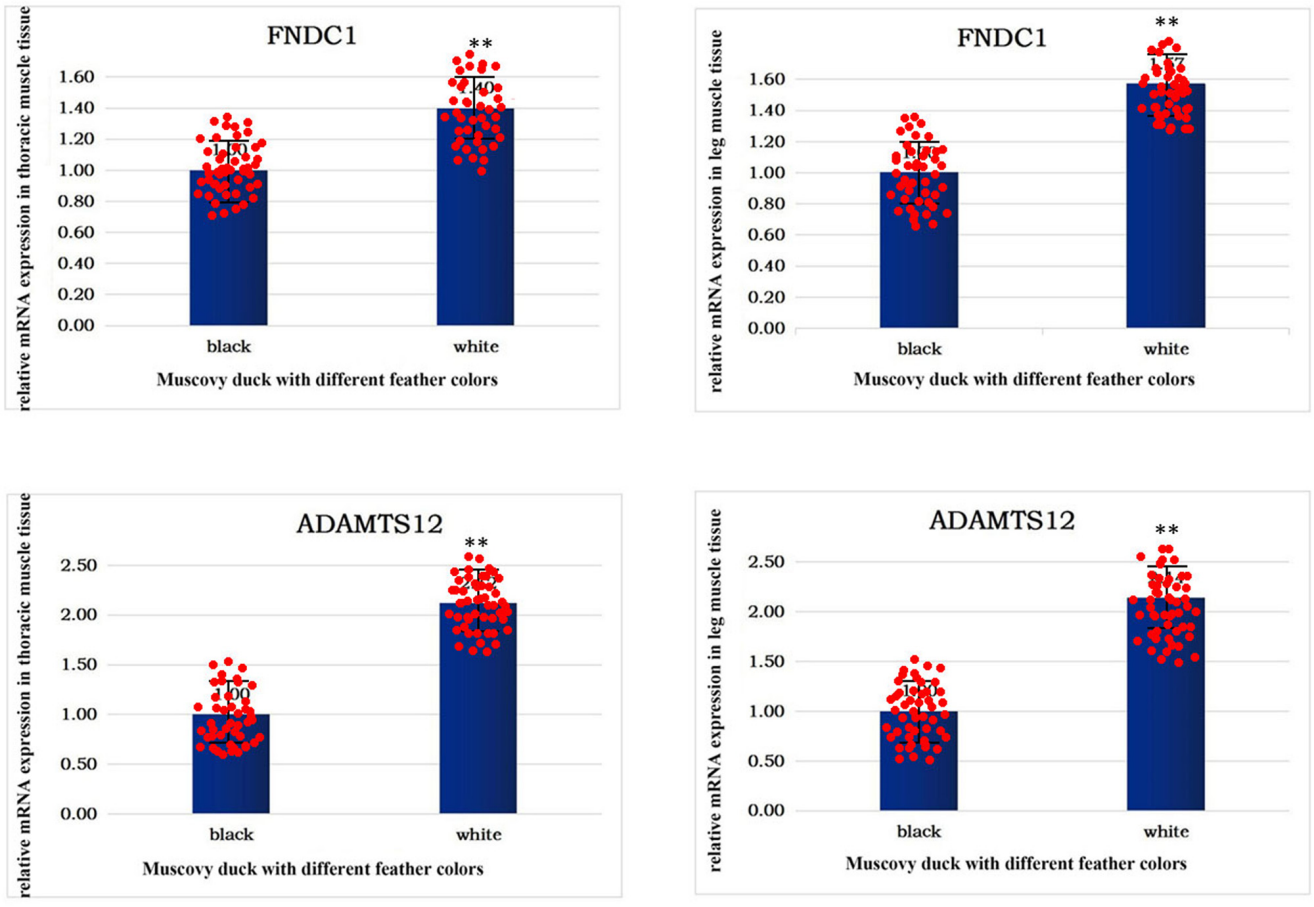
mRNA expression level of *FNDC1* and *ADAMTS12*. **Compared with black. *P* < 0.01.

In this research, we investigated the mRNA expression patterns of the *FNDC1* and *ADAMTS12* genes within the muscle tissues of Muscovy ducks characterized by distinct plumage colors: black and white. The outcomes,as depicted in Figure 2, the expression levels of both *FNDC1* and *ADAMTS12* genes in Muscovy ducks with white feathers was significantly higher than Muscovy ducks with black feathers. This pivotal finding illuminates the genetic underpinnings of feather color variations in Muscovy ducks, providing crucial insights into avian genetic diversity and evolutionary dynamics. Furthermore, these insights hold practical significance for poultry breeding endeavors, potentially facilitating the selective breeding of ducks with preferred feather colors. Such advancements could significantly contribute to the poultry industry’s economic value by enabling the customization of plumage color in domestic ducks.

#### 3.2.2 Protein expression results

In this study, we performed an in-depth examination of muscle tissues from black and white Muscovy ducks to explore the protein expression profiles of the *FNDC1* and *ADAMTS12* genes. The results, presented in Table 2, distinctly underscore the differential patterns of gene expression between the two color phenotypes. Specifically, Muscovy ducks with purely white feathers demonstrated significantly higher expression levels of the *FNDC1* gene, while those with purely black feathers showed diminished expression levels of the *ADAMTS12* gene relative to their white-feathered counterparts.

**Table 2:** Findings from the analysis of protein expression in pertinent genes

Gene name	Expression quantity	
	Pure black feather Muscovy ducks	Pure white feather Muscovy ducks
FNDC1	1.13 ± 0.11	1.18 ± 0.15
ADAMTS12	1.09 ± 0.09	1.07 ± 0.10

## 4 Discussion

The expression of the *FNDC1* gene plays a pivotal role in the coloration and patterning of feathers in poultry, influencing both the deposition of pigments and the differentiation of pigment cells. Meanwhile, *ADAMTS12*, functioning as a metalloproteinase, is involved in the metabolism and regulatory processes of various extracellular matrices. Through its regulation of collagen synthesis and degradation, *ADAMTS12* impacts the structural organization and tissue configuration of the skin matrix. This, in turn, affects the distribution of pigment cells and the formation of pigment deposits, thereby contributing to the observed variations in feather color and patterns.

The regulation of feather color represents a complex trait influenced by the concerted action of multiple genes, epitomizing a trait characterized by polygenic causation leading to a singular phenotypic effect [5]. Presently, the focus of research efforts has been on exploring variations in feather color and examining the genetic underpinnings of feather coloration in Muscovy ducks [6]. Despite these efforts, there is a scarcity of literature dedicated to unraveling the genetic mechanisms and the correlation between molecular expressions related to feather color in Muscovy ducks. Additionally, there is a conspicuous gap in research concerning gene transcriptomics, highlighting an area ripe for further investigation.

Fibronectin (FN) is a high molecular weight adhesive glycoprotein predominantly occurring in dimeric form [7]. It is ubiquitously present across animal tissues and bodily fluids, fulfilling several critical biological functions [7,8]. *FNDC1*, classified as a type III FN gene situated on the human chromosome 6q25.3, acts as a receptor-independent facilitator of G protein signal transduction. This action is mediated through its interaction with the Gβγ subunit, thereby initiating G protein signal transduction pathways [9].

Prior studies have established a correlation between the expression of *FNDC1* and the occurrence of various human diseases, including gastric cancer and a range of other tumorous conditions [9,10]. Within the context of poultry, particularly in broilers, *FNDC1* expression has been linked to femoral head separation, a condition affecting growth [11]. Despite these associations, current literature lacks information regarding the potential relationship between *FNDC1* gene expression and feather traits in Muscovy ducks.

A Disintegrin And Metalloproteinase with Thrombospondin Motifs (ADAMTS) represents a family of zinc-dependent proteases found in both invertebrates and mammals, characterized by their function as depolymerizing, protein-like metalloproteinases, which include a type I platelet-binding protein motif. Comprising 19 distinct members, this group shares the type I platelet-binding protein motifs with ADAM metalloproteinases and matrix metalloproteinases, situating them within the broader family of secreted enzymes known as metalloproteinases. These enzymes are instrumental in modulating the structural and functional dynamics of the extracellular matrix, as well as the regulation of extracellular proteins circulating within the bloodstream [12].

The ADAMTS enzyme family is classified into various subgroups, distinguished by their distinct structural features, with each subgroup fulfilling specific biological roles. For instance, *ADAMTS7* and *ADAMTS12* are unique in that they contain a mucin domain within the thrombospondin repeat sequence at their C-terminal end, a characteristic that facilitates their ability to cleave cartilage proteins [13]. On another front, *ADAMTS15* is recognized for its function as a tumor suppressor gene, playing a pivotal role in tumor development [14]. Similarly, the expression of *ADAMTS13* has been associated with the incidence of cardiovascular diseases, including stroke [12]. In an avian study, Carré et al. demonstrated the role of *ADAMTS12* in the process of gonad formation in birds [15]. Concurrently, Guo et al. [16] proposed a potential connection between *ADAMTS3* gene expression and the physical traits of combs in domestic chickens. However, there is a notable absence of research on the involvement of the ADAMTS family in determining the plumage of Muscovy ducks.

In our research, we explored the mRNA expression and protein concentrations of the *FNDC1* and *ADAMTS12* genes to ascertain their correlation with feather color variations in Muscovy ducks. The findings demonstrated differential expression levels of these genes associated with feather color. Notably, both *FNDC1* and *ADAMTS12* genes exhibited significantly elevated expression in Muscovy ducks possessing white feathers in comparison to their counterparts with black feathers.

Furthermore, among the pure white Muscovy ducks, the expression level of the *FNDC1* gene was significantly elevated compared to that in the pure black Muscovy ducks. Conversely, the expression of the *ADAMTS12* gene was found to be lower in pure black ducks when compared with pure white ducks. Discrepancies between the mRNA and protein levels of the *FNDC1* and *ADAMTS12* genes were observed, indicating the possibility of regulatory interferences affecting their expression. This may play a role in the observed variations in feather color across different anatomical locations.

Our results shed light on the potential genetic factors underlying feather color variations in Muscovy ducks, delivering crucial contributions to the field of avian genetics. Additionally, the implications of this research extend into practical fields, furnishing valuable data for selective breeding initiatives focused on optimizing particular characteristics within domestic duck populations. Such advancements hold the promise of conferring substantial benefits upon the poultry industry, by facilitating the development of breeds with desired phenotypic traits.

The insights garnered from this study underscore the need for further investigation to enhance our understanding significantly. To achieve a more comprehensive grasp of the roles played by the *FNDC1* and *ADAMTS12* genes in dictating feather color traits in Muscovy ducks, it is imperative to extend the research in several key areas. Increasing the sample size will provide a broader data set for analysis, thereby enhancing the statistical power and reliability of the findings. Advancing the research methodologies used will allow for more precise and accurate measurement of gene expression and function. Additionally, validating the expression patterns and regulatory mechanisms of the *FNDC1* and *ADAMTS12* genes through further experimental studies is crucial for confirming their influence on feather color variations.

## 5 Conclusion

In this study, we identified a significant correlation between the *FNDC1* and *ADAMTS12* genes and feather color variations in Muscovy ducks. However, to fully elucidate the exact mechanisms of their expression and the regulatory pathways involved, further detailed investigations are necessary.
